# Impact of vaccination on new SARS-CoV-2 infections in the United Kingdom

**DOI:** 10.1038/s41591-021-01410-w

**Published:** 2021-06-09

**Authors:** Emma Pritchard, Philippa C. Matthews, Nicole Stoesser, David W. Eyre, Owen Gethings, Karina-Doris Vihta, Joel Jones, Thomas House, Harper VanSteenHouse, Iain Bell, John I. Bell, John N. Newton, Jeremy Farrar, Ian Diamond, Emma Rourke, Ruth Studley, Derrick Crook, Tim E. A. Peto, A. Sarah Walker, Koen B. Pouwels

**Affiliations:** 1 Nuffield Department of Medicine, University of Oxford, Oxford, UK; 2 National Institute for Health Research (NIHR) Health Protection Research Unit in Healthcare Associated Infections and Antimicrobial Resistance, University of Oxford, Oxford, UK; 3 NIHR Oxford Biomedical Research Centre, University of Oxford, Oxford, UK; 4 Department of Infectious Diseases and Microbiology, Oxford, University Hospitals NHS Foundation Trust, John Radcliffe Hospital, Oxford, UK; 5 Big Data Institute, Nuffield Department of Population Health, University of Oxford, Oxford, UK; 6 Office for National Statistics, Newport, UK; 7 Department of Mathematics, University of Manchester, Manchester, UK; 8 IBM Research, Hartree Centre, Daresbury, UK; 9 Glasgow Lighthouse Laboratory, Glasgow, UK; 10 BioClavis, Glasgow, UK; 11 Office of the Regius Professor of Medicine, University of Oxford, Oxford, UK; 12 Health Improvement Directorate, Public Health England, London, UK; 13 Wellcome Trust, London, UK; 14 MRC Clinical Trials Unit at UCL, University College London, London, UK; 15 Health Economics Research Centre, Nuffield Department of Population Health, University of Oxford, Oxford, UK

**Keywords:** Viral infection, Epidemiology, Outcomes research

## Abstract

The effectiveness of COVID-19 vaccination in preventing new severe acute respiratory syndrome coronavirus 2 (SARS-CoV-2) infections in the general community is still unclear. Here, we used the Office for National Statistics COVID-19 Infection Survey—a large community-based survey of individuals living in randomly selected private households across the United Kingdom—to assess the effectiveness of the BNT162b2 (Pfizer–BioNTech) and ChAdOx1 nCoV-19 (Oxford–AstraZeneca; ChAdOx1) vaccines against any new SARS-CoV-2 PCR-positive tests, split according to self-reported symptoms, cycle threshold value (<30 versus ≥30; as a surrogate for viral load) and gene positivity pattern (compatible with B.1.1.7 or not). Using 1,945,071 real-time PCR results from nose and throat swabs taken from 383,812 participants between 1 December 2020 and 8 May 2021, we found that vaccination with the ChAdOx1 or BNT162b2 vaccines already reduced SARS-CoV-2 infections ≥21 d after the first dose (61% (95% confidence interval (CI) = 54–68%) versus 66% (95% CI = 60–71%), respectively), with greater reductions observed after a second dose (79% (95% CI = 65–88%) versus 80% (95% CI = 73–85%), respectively). The largest reductions were observed for symptomatic infections and/or infections with a higher viral burden. Overall, COVID-19 vaccination reduced the number of new SARS-CoV-2 infections, with the largest benefit received after two vaccinations and against symptomatic and high viral burden infections, and with no evidence of a difference between the BNT162b2 and ChAdOx1 vaccines.

## Abstract (web summary)

Results from the Office of National Statistics COVID-19 Infection Survey in the United Kingdom demonstrate that the ChAdOx1 nCoV-19 and BNT162b2 vaccines reduce the incidence of new SARS-CoV-2 infections by up to 65% with a single dose and up to 80% after two doses, with no significant differences in efficacy observed between the two vaccines.

## Main

On 8 December 2020, the United Kingdom was the first country to start a COVID-19 vaccination program following emergency use authorization of the BNT162b2 messenger RNA (mRNA) vaccine (Pfizer–BioNTech) by the United Kingdom’s Medicines and Healthcare Products Regulatory Agency^1^. Additional COVID-19 vaccines have since been approved, including the Oxford–AstraZeneca adenovirus vector vaccine, ChAdOx1 nCOV-19 (termed here ChAdOx1)^2^, and more recently an mRNA-based COVID-19 vaccine developed by Moderna, mRNA-1273 (ref. ^3^). To date, most vaccinated individuals in the United Kingdom have received one or two doses of the BNT162b2 or ChAdOx1 vaccines, which are the vaccines focused on in the current study.

Initially, those in care homes, those over 80 years old and frontline health and social care workers were prioritized for vaccination^4^. Clinically vulnerable people and those aged ≥70 years were the next priority groups, followed by remaining adults in order of decreasing age. As of 14 April, over 32 million (62%) UK adults (≥18 years old) had received at least one COVID-19 vaccine dose^5^, most of whom had received one dose only following the extension of the dosing interval to 12 weeks to maximize initial coverage^6^. UK inhabitants were invited to receive a COVID-19 vaccine independent of antibody status, although those testing PCR positive just before their scheduled vaccination had to reschedule their appointment to a later date to minimize the chances of an outbreak at vaccination sites.

Large randomized trials estimated the efficacy against symptomatic laboratory-confirmed COVID-19 infection to be 70% (95% confidence interval (CI) = 55–81%) after two ChAdOx1 doses^7^ and 95% (95% CI = 90–98%) after two BNT162b2 doses^8^. While trials provide unbiased effect estimates, trial participants may differ from the general population in many ways, so it is essential to assess effectiveness in the community, particularly given differences between real-world vaccine deployment and the licensed dosing schedule. Comparing vaccine effectiveness in the community is also important as the trials used different outcome definitions (for example, the start of the at-risk period was 14 d (ref. ^7^) versus 7 d (ref. ^8^) after the second dose for the ChAdOx1 and BNT162b2 vaccine trials, respectively) and populations (for example, there was a smaller proportion of participants aged >55 years in the ChAdOx1 vaccine trial (12%)^7^ versus the BNT162b2 vaccine trial (42%)^8^).

Furthermore, both trials were largely conducted before the severe acute respiratory syndrome coronavirus 2 (SARS-CoV-2) variant B.1.1.7 became dominant in the United Kingdom^9^. This variant is more transmissible and potentially causes more severe disease^10–12^. Concerns have been raised that some of its defining mutations may affect the efficacy of vaccines and natural infection-derived immunity to (re)infection^13^. A subset of 8,534 participants from the initial ChAdOx1 trial were followed for a longer period to assess protection against different viral variants, but wide CIs meant it was difficult to conclude whether the efficacy was lower against B.1.1.7 (70% (95% CI = 44–85%) than other lineages (82% (95% CI = 70–89%))^14^.

Ongoing assessment of the effectiveness of different vaccines across different subgroups is critical, especially among older adults, who were under-represented in the ChAdOx1 trials. Real-world studies are starting to appear, with an analysis from Israel estimating 92% (95% CI = 88–95%) effectiveness against symptomatic PCR-confirmed infection ≥7 d after the second BNT162b2 dose^15^. A study among healthcare workers in England found an effectiveness of 70% (95% CI = 55–85%) 21 d after a first dose and 85% (95% CI = 74–96%) after a second dose of BNT162b2 against PCR-positive infections^16^. Another study assessing the early effectiveness of the BNT162b2 and ChAdOx1 vaccines in older adults (≥70 years) in England showed that a single dose of either vaccine was ~60% effective against symptomatic laboratory-confirmed infection and ~80% effective against hospitalization^17^. The evidence on effectiveness against asymptomatic infection is limited, with one study among 13,109 healthcare workers from Oxfordshire, United Kingdom, showing a 64% (95% CI = 50–74%) reduction in any SARS-CoV-2 PCR-positive result following a single BNT162b2 or ChAdOx1 dose^9^. Another study among 3,950 healthcare workers, first responders and other essential and frontline workers from the United States estimated 80% (95% CI = 59–90%) and 90% (95% CI = 68–97%) vaccine effectiveness 14 or more days after one or two doses of the BNT162b2 or mRNA-1273 vaccine, respectively^18^. Most recently, a study of 10,412 residents of long-term care facilities showed 65 and 68% protection against SARS-CoV-2 PCR-positive results 28–42 d after vaccination with the ChAdOx1 or BNT162b2 vaccine, respectively^19^.

However, existing studies have either investigated defined sub-populations^9,18,19^ or have relied on the results from symptomatic testing programs^15,17^, potentially leading to bias from vaccination status influencing the test-seeking behavior of cases not requiring health care. Large community-based studies where testing is done in a systematic manner (independent of both vaccination status and symptoms) are lacking. We therefore used the Office for National Statistics (ONS) COVID-19 Infection Survey—a large community-based survey of individuals aged 2 years and older living in randomly selected private households across the United Kingdom—to assess the effectiveness of the BNT162b2 and ChAdOx1 vaccines, as implemented in the United Kingdom, against any SARS-CoV-2 PCR-positive test performed in the survey^20^, where real-time PCR (RT-PCR) tests were performed on a fixed schedule, irrespective of symptoms, vaccine status and previous infection. We assessed vaccine effectiveness based on overall RT-PCR positivity and split according to self-reported symptoms, cycle threshold (Ct) value (<30 versus ≥30; as a surrogate for viral load) and gene positivity pattern (compatible with B.1.1.7 or not).

## Results

### Characteristics of visits and new PCR positives included in analysis

From 1 December 2020 to 8 May 2021, 383,812 individuals from 216,953 households provided 1,945,071 RT-PCR results from nose and throat swabs in the COVID-19 Infection Survey (median (interquartile range (IQR)) = 5 (4–6)), of which 12,826 (0.8%) were the first positive in an infection episode and 1,932,245 (99.3%) were negative. The characteristics at each visit when these swabs were taken, and hence included in the analyses, are shown in Supplementary Table 1. The median (IQR) age at included visits was 55 years (40–68 years). Of the total swab results, 6% were from those reporting non-white ethnicity, 4% were from those reporting patient-facing health/social care work or working in a care home (high-priority group for vaccination) and 27% were from those reporting a long-term health condition (priority group for vaccination).

**Extended Data Fig. 1:**
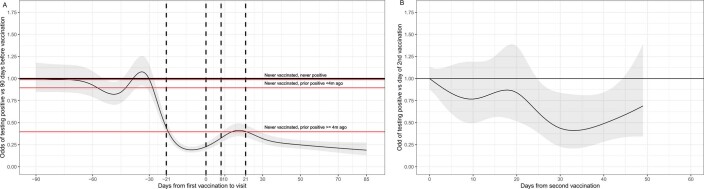
Estimated effect of days since from vaccination on odds of testing positive on a continuous scale. **a**, Days from first vaccination to visit. Note: arbitrarily categorised in main analysis at dashed lines as shown. Odds ratios were obtained from a generalised linear model with logit link with 90 days before vaccination as the reference time for the spline used for time since first vaccination (panel A), and the day of the second vaccination as the reference time for the spline for time since second vaccination (Panel B). **b**, Days from second vaccination to visit.

We classified each visit according to vaccination status and previous infection (Supplementary Table 2), classifying the time from vaccination empirically based on modeling the days since the first vaccination as a continuous nonlinear effect (Extended Data Fig. 1). The baseline group included visits occurring >21 d before vaccination in those with no evidence of previous infection (1,012,808 visits; 10,721 new PCR positives). A further 21,442 visits (105 PCR positives) occurred in unvaccinated participants with evidence of previous infection, with a median of 125 d (IQR = 106–161 d) from the first positive (study antibody, study swab or external test in the national program) to the included visit. As <4% of visits in each vaccinated group occurred in individuals with evidence of previous infection before vaccination (Supplementary Table 2), visits were classified based on vaccination history alone. As there were insufficient data to estimate the effects of vaccination dependent on previous infection status, we therefore estimated the effectiveness of vaccination as implemented in the United Kingdom. In total, 137,575 visits (95 PCR positives) occurred a median of 16 d (IQR = 7–30) after a second dose; among the 99,267 individuals who received a second dose, the median number of days between the first and second dose was 73 (IQR = 63–77 d).

**Fig. 1:**
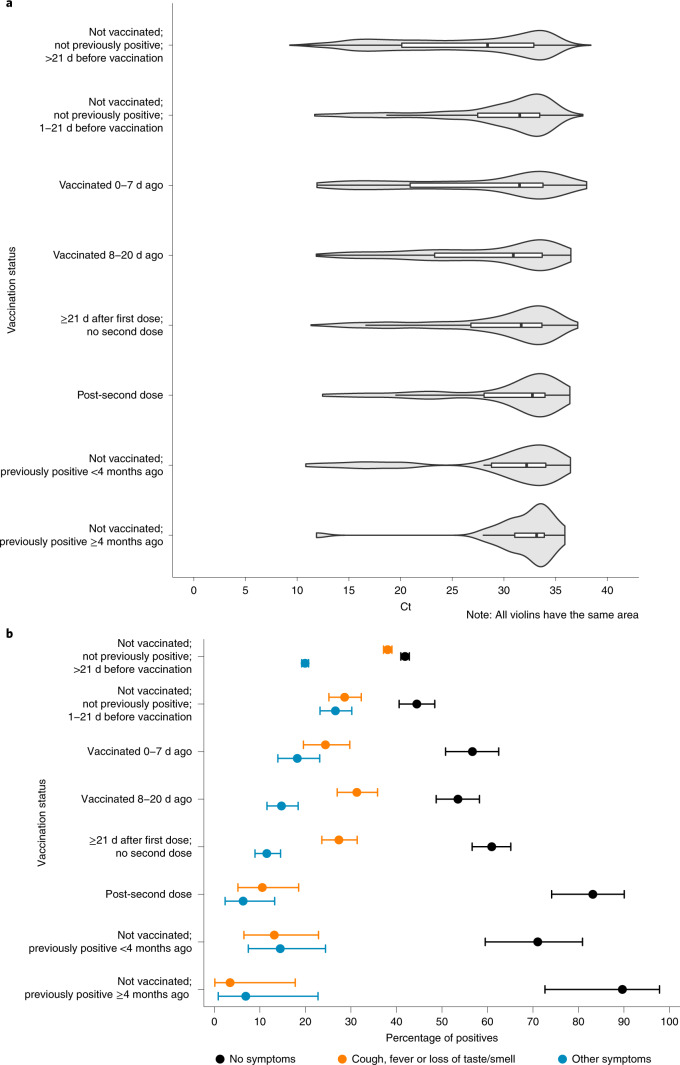
Distribution of Ct values and percentage of symptoms in new positive episodes by vaccination status. **a**, Distribution of Ct values. **b**, Percentage of symptoms. The numbers of visits with a positive test contributing to the plots by exposure group were: 10,721 (not vaccinated; not previously positive; >21 d before vaccination); 643 (not vaccinated; not previously positive; 1–21 d before vaccination); 291 (vaccinated 0–7 d ago); 441 (vaccinated 8–20 d ago); 530 (≥21 d after first dose; no second dose); 95 (post-second dose); 76 (not vaccinated; previously positive <4 months ago); and 29 (not vaccinated; previously positive ≥4 months ago). Boxplots inside violin plots in **a** show median values and upper and lower quartiles of the distribution, with whiskers extending from the hinge to the largest and smallest value no further than 1.5 times the IQR. The error bars in **b** represent 95% CIs. Values are given in Supplementary Table 3.

In new infections, Ct values (inversely related to viral load) increased with increasing time from the first vaccination as well as the number of doses (Fig. 1a and Supplementary Table 3). The highest Ct values were in those who had received two vaccine doses, with a similar distribution to those not vaccinated but previously PCR or antibody positive. Ct values were lowest in those not vaccinated and not previously PCR or antibody positive.

The percentage of PCR-positive individuals self-reporting symptoms was highest in those not vaccinated and not previously PCR or antibody positive (58% at >21 d before vaccination) and lowest in those with two vaccine doses (17%) and those not vaccinated but previously PCR or antibody positive ≥4 months before (10%; Fig. 1b). Well-recognized COVID-19 symptoms (cough, fever and loss of taste or smell) were most commonly reported in unvaccinated individuals who were not previously PCR or antibody positive.

### Impact of any COVID-19 vaccination on new infections

**Fig. 2:**
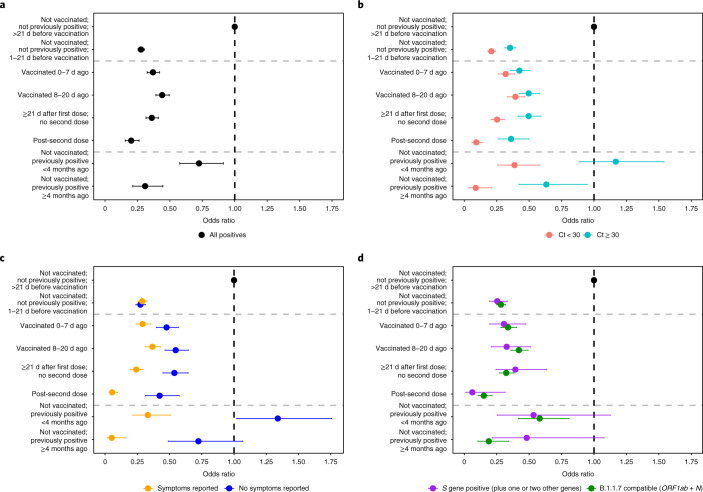
Adjusted odds ratios for the effect of vaccination and previous positivity on all positives and positives split by Ct score, self-reported symptoms and gene positivity pattern. **a**–**d**, Adjusted odds ratios for effects on all positives (**a**) and positives split by Ct value (**b**), self-reported symptoms (**c**) and gene positivity pattern (**d**). All odds ratios were obtained from a generalized linear model with a logit link comparing each category with the reference category (not vaccinated; not previously positive; >21 d before vaccination) and using clustered robust standard errors. Odds ratios are given in Supplementary Table 4. The numbers of visits underlying the models for the different outcomes are provided in Supplementary Table 8. All error bars represent 95% CIs.

**Extended Data Fig. 2:**
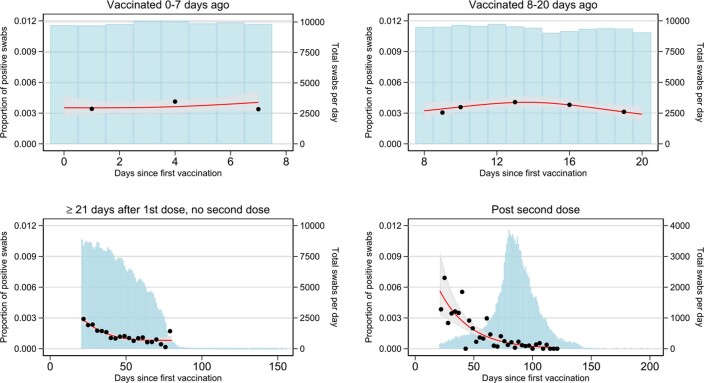
Observed proportion of positives and numbers of visits over days from vaccination. Note: observed proportion of positives grouped over every 3 days since vaccination (black dots) with fit of restricted natural cubic spline (fit to each study day) with 3 knots at the 10^th^,50^th^ and 90^th^ percentile of the unique values of study day (red line) and 95% confidence intervals. Number of individuals on each vaccination day (denominator of the proportions) is shown by the blue bars.

In unadjusted analyses, the percentage of positive PCR tests remained stable over the first 20 d following vaccination but decreased from 21 d onwards regardless of whether one or two doses had been administered (Extended Data Fig. 2). Adjusting for multiple potential confounders, the vaccine effectiveness ((1 − odds ratio) × 100) against new PCR positives, with or without symptoms, was 56% (95% CI = 51–61%) in those 8–20 d after vaccination versus the baseline group, with no evidence of a difference versus those vaccinated 0–7 d ago (*P* = 0.251). The vaccine effectiveness was 64% (95% CI = 59–68%; *P* < 0.001) in those assessed ≥21 d since the first vaccination with no second dose—marginally higher than in those vaccinated 8–20 d ago (*P* = 0.066) (Fig. 2a and Supplementary Table 4; coefficients for all factors in Supplementary Table 5). The odds of testing positive were reduced to 72% (95% CI = 70–75%) 1–21 d before the first vaccination and 63% (95% CI = 58–68%) 0–7 d post-vaccination versus the baseline group.

The largest vaccine effectiveness was estimated among those following the second vaccine dose (80%; 95% CI = 74–84%; *P* < 0.001), and this was significantly greater than for those having received only one dose ≥21 d previously (*P* < 0.001). There was no evidence that reductions in the odds of testing positive differed between those having received two vaccine doses and those not having been vaccinated but who were PCR or antibody positive >4 m previously (*P* = 0.523) (Supplementary Table 4).

The benefits associated with vaccination were much greater for infection episodes with Ct < 30 (as evidence of high levels of viral shedding) compared with Ct ≥ 30 (Fig. 2b), with the vaccine effectiveness against testing positive with Ct < 30 estimated at 91% (95% CI = 85–94%; *P* < 0.001) post-second dose—a greater benefit compared with the 75% (69–79%) effectiveness following one dose ≥21 d ago (*P* < 0.001) and compared with no evidence of a difference versus those not vaccinated but PCR or antibody positive >4 m previously (*P* = 1.00). Similarly, the benefits associated with vaccination were much greater for self-reported symptomatic infection episodes (Fig. 2c), with an estimated vaccine effectiveness against testing positive with self-reported symptoms of 95% (95% CI = 90–97%; *P* < 0.001) post-second dose—significantly higher than with one dose ≥21 d ago (*P* < 0.001) (Supplementary Table 4), but again without evidence of a difference versus those not vaccinated but PCR or antibody positive >4 m previously (*P* = 1.00). In comparison, the estimated vaccine effectiveness against new infection episodes with no self-reported symptoms was 58% (95% CI = 43–69%; *P* < 0.001) post-second dose. While some of the cases overlapped, positives with Ct < 30 also differed from positives where symptoms were reported; for example, 4,731 (37%) of all positives had Ct < 30 and symptoms reported, whereas 2,125 (17%) had Ct < 30 and no symptoms reported (Supplementary Table 3). The effects of vaccination on infections compatible and not compatible with the B.1.1.7 variant appeared similar, but small numbers of positives in the latter group led to large uncertainty in the estimates (Fig. 2d and Supplementary Table 4).

### Impact of vaccination type on new infections

**Fig. 3:**
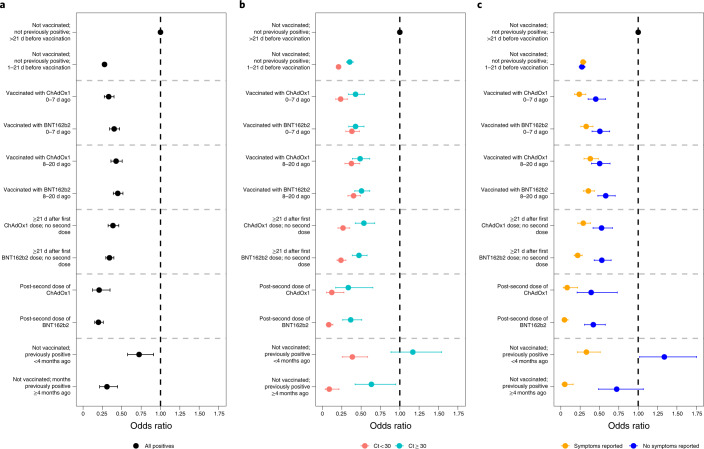
Adjusted odds ratios for the effect of vaccination, split by vaccine type and previous positivity, on all positives and positives split by Ct value and self-reported symptoms. **a**–**c**, Adjusted odds ratios for effects on all positives (**a**) and positives split by Ct value (**b**) and self-reported symptoms (**c**). All odds ratios were obtained from a generalized linear model with a logit link comparing each category with the reference category (not vaccinated; not previously positive; >21 d before vaccination) and using clustered robust standard errors. Odds ratios are given in Supplementary Table 6. The numbers of participants and visits underlying the models for the different outcomes are provided in Supplementary Table 9. All error bars represent 95% CIs.

There was no evidence that the vaccine effectiveness against new infections differed between the BNT162b2 and ChAdOx1 vaccines (Fig. 3a and Supplementary Table 6), whether the vaccine was received 0–7 d previously (*P* = 0.799), 8–20 d previously (*P* = 1.00), ≥21 d previously (*P* = 0.940) or post-second dose (*P* = 0.709). At ≥21 d after the first dose, the effectiveness of the BNT162b2 and ChAdOx1 vaccines was 66% (95% CI = 60–71%) versus 61% (95% CI = 54–68%), respectively. After two doses, the effectiveness was 80% (95% CI = 73–85%) versus 79% (95% CI = 65–88%), respectively. There was also no evidence that reductions in the odds of new infections differed between those post-second BNT162b2 dose and those not vaccinated but PCR or antibody positive >4 m previously (*P* = 0.704). The effects were similar considering infections with Ct < 30 versus ≥30 (Fig. 3b) or with versus without self-reported symptoms (Fig. 3c), with the impact of both vaccines attenuated to similar degrees for infections with Ct ≥ 30 and without self-reported symptoms.

### Potential subgroup effects

**Fig. 4:**
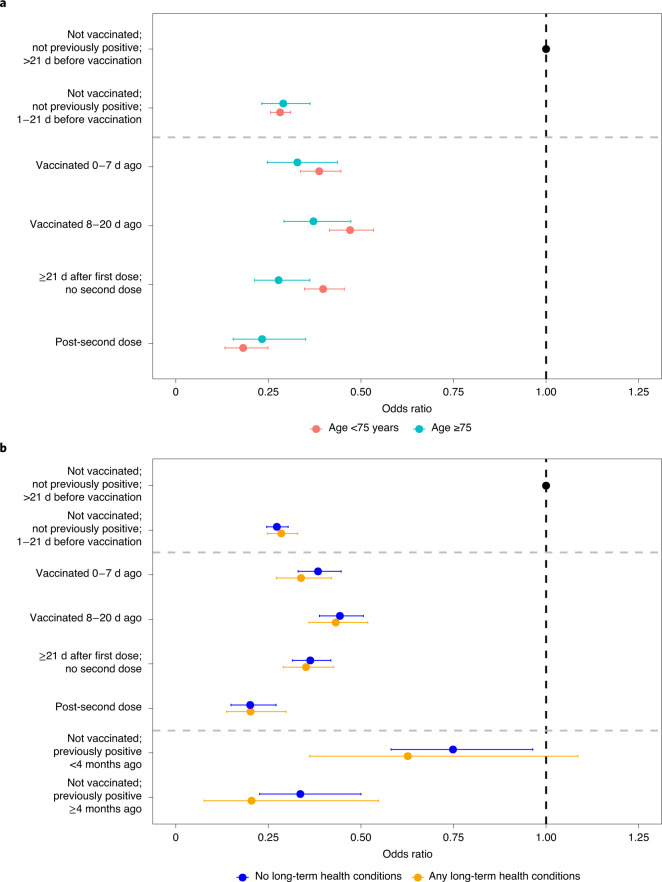
Adjusted odds ratios for the effect of vaccination, split by age and long-term health conditions, on all positives. **a**,**b**, Adjusted odds ratios for effects on all positives, split by age (**a**) and long-term health conditions (**b**). All odds ratios were obtained from a generalized linear model with a logit link comparing each category with the reference category (not vaccinated; not previously positive; >21 d before vaccination) and using clustered robust standard errors. The numbers of participants and visits in the different subgroups are provided in Supplementary Table 10a (by age, corresponding to **a**) and Supplementary Table 10b (by the presence or absence of long-term health conditions, corresponding to **b**). The heterogeneity *P* values (as determined by two-sided Wald test) for the two vaccination categories were: *P* = 0.011 (age) and *P* = 0.897 (long-term health conditions). There were no positives in those aged ≥75 years in the previously infected exposure groups, so these groups were excluded from the subgroup analysis by age. All error bars represent 95% CIs.

There was evidence of differences in the effects of vaccination on new infections between those aged under and over 75 years (global heterogeneity for all vaccination terms *P* = 0.011; Fig. 4a). This was driven by greater benefits in those ≥21 d since the first vaccination with no second dose, where reductions in the odds were 72% in those aged ≥75 years (95% CI = 64–78%) and 60% in those <75 years (95% CI = 54–65%) (interaction *P* = 0.007). There was no evidence of differences in the effect of vaccination on new infection between those reporting or not reporting long-term health conditions (global heterogeneity for all vaccination terms *P* = 0.897).

### Sensitivity analyses

While most potential confounders are unlikely to have been affected by vaccination itself, work location, mode of travel to work and contacts with care homes, including visiting relatives, could theoretically be both confounders and affected through vaccination and previous infections through risk compensation. Sensitivity analyses excluding these factors resulted in very similar estimates of vaccine effectiveness ≥21 d since the first vaccination with no second dose (64% (95% CI = 59–68%) versus 60% (95% CI = 54–65%)), as well as post-second dose (80% (95% CI = 74–83%) versus 77% (95% CI = 70–82%)) (Supplementary Table 7).

The Ct threshold of <30 versus ≥30 was selected on the basis of it being used in the United Kingdom in algorithms for the review of low-level positives at the laboratories where the PCR tests were performed and as a threshold for attempting whole-genome sequencing. We also performed a sensitivity analysis with an arbitrary threshold of Ct < 25 versus Ct ≥ 25 and found that this increased the estimated vaccine effectiveness against low- and high-Ct infections, as lowering this threshold shifts both groups to a lower Ct value (higher viral load). For example, the estimated effectiveness ≥21 d after the first dose was 79% (95% CI = 73–84%) for Ct < 25 and 55% (95% CI = 47–61%) for Ct ≥ 25, while these values were 75% (95% CI = 69–79%) and 50% (41–58%) for Ct < 30 and Ct ≥ 30, respectively.

## Discussion

The results from this large community surveillance study show that vaccination against COVID-19, with either the ChAdOx1 vaccine or the BNT162b2 vaccine, substantially reduced the odds of individuals testing PCR positive with a new SARS-CoV-2 infection, with the greatest reductions in new infections observed in individuals with Ct < 30 and self-reported symptoms, and in those who had received two vaccine doses. Reductions afforded by vaccination were similar to those in individuals who were not vaccinated but had been PCR or antibody positive >4 months previously. The protective effect of vaccination was more pronounced in infections with Ct < 30 and with self-reported symptoms. There was no evidence of any difference in effectiveness between the BNT162b2 and ChAdOx1 vaccines, nor in those with long-term health conditions. We observed greater reductions in new infections in those aged ≥75 years versus those under 75 years after one dose, but this difference was not apparent after two doses.

The main study strength is its design as a large-scale community survey recruiting from randomly selected private residential households, providing a representative sample of the UK general population. Participants were tested regardless of symptoms, allowing us to additionally consider vaccine effectiveness against infection without reported symptoms. The availability of Ct values allowed us to compare the vaccine impact on viral loads, using Ct as a proxy^21^. Scheduled visits provided an unbiased sampling frame, which we exploited for our logistic regression (rather than having to censor individuals at the last tests in the study using time-to-event analyses and assuming that all infections between visits were identified). Participants were asked about demographics, behaviors and work, allowing us to control for a wide range of potential confounders that are unavailable in record linkage studies performed to date^15^.

The study design also had limitations, particularly with individuals tested initially at weekly and then monthly visits. Vaccination status was based on self-reporting in Northern Ireland, Wales and Scotland, potentially leading to some exposure misclassification. However, the vast majority of visits were for people living in England, where there was good agreement between self-reported and administrative vaccination data (98% on type and 95% on date). Antibody status was only measured in a subsample of study participants, meaning that some participants infected before joining the survey or not detected at survey visits will have been misclassified as having no previous antibody-positive test. Similarly, any positive episodes occurring between visits and not captured by the national testing program will have been missed, leading to contamination of the group that was not vaccinated and had no previous PCR- or antibody-positive test, possibly diluting the observed effects of vaccination. We used national testing program positives only for exposure classification, to avoid the potential bias due to testing behavior being influenced by vaccination and previous infection status. However, because participants could therefore only have a new positive outcome at scheduled visits, some of the new positives episodes could have occurred sometime previously; we therefore stratified the time from vaccination to reduce the impact of this. Older infections would be expected to have higher Ct values, which might partly explain the differences between positives with a Ct value of <30 versus ≥30, at least shortly after vaccination. The imperfect sensitivity of SARS-CoV-2 PCR tests may also have biased the absolute risk, but would result in an unbiased relative risk provided that outcome misclassification was non-differential to vaccination status and all non-cases were correctly classified (that is, 100% specificity). The PCR test specificity was probably very high (>99.99%)^12,20^; therefore, any bias here is expected to be small. Due to relatively small numbers of infections post-vaccination, the power to detect differences between vaccine types and differential vaccine effectiveness in subgroups was relatively low.

An important potential issue with observational studies evaluating vaccine effectiveness is that individuals are not supposed to be vaccinated if they have tested positive in the past 4 weeks^22^, and individuals may reduce their number of contacts in response to the knowledge that they will soon receive a vaccination. We found that 643 individuals tested positive 1–21 d before receiving their vaccination (due to the design and logistics of the survey, they might have received their test results after the date of vaccination), suggesting that ensuring social distancing at vaccination locations remains important. Rather than representing a biological effect, the reduced risk observed in the 21 d before and 0–7 d after vaccination could be due to this reverse causality (specifically, changes in behavior due to either receiving the vaccination invitation letter, knowledge that individuals from the same age or risk group are about to receive a vaccination in their area or postponement of a planned vaccination visit due to a positive COVID-19 test in the 28 d before their scheduled vaccination appointment). In the hypothetical situation where all infections are detected immediately and adherence to guidance is perfect, there would be zero new infections observed 1–21 d before vaccination, emphasizing the large temporary impact that reverse causality can have. In theory, an unmeasured confounder could also explain the reduction in positive tests 1–21 d before receiving a vaccination compared with >21 d before receiving a vaccination; however, this would need to be a strong time-varying confounder that is at most weakly associated with calendar time, as the latter was included in the model using a flexible tensor spline that modeled the interaction between nonlinear effects of age and calendar time, and was also allowed to vary by region/country. Given this, it is much more likely that reverse causality underlies the lower odds of positive tests before and shortly after vaccination. Because a reduction in contacts and acquisition of infections in the week before vaccination will also reduce the likelihood of testing positive in the following week, it will be important for future studies aiming to evaluate the effectiveness of vaccination to carefully construct the appropriate comparator. Here, we used study visits for those who were not vaccinated and not previously positive, at ≥21 d before vaccination, as the baseline group to overcome these issues when estimating the impact of vaccination.

Our estimated effect of two vaccine doses on symptomatic infections is similar to that in the key phase III clinical trials^7,8^ but slightly higher than other non-randomized studies that have considered this outcome but focused on specific populations or were potentially affected by changes in test-seeking behavior associated with vaccination^9,15,17–19^. Higher Ct values in infections identified post-vaccination have also been demonstrated in older adults in care homes^19^. Our estimated reduction in the risk of new PCR positives for those not vaccinated but infected >4 m previously (69%) was slightly lower than the ~80% (95% CI = 75.4–84.5%) estimated elsewhere^23^, but we observed greater reductions against symptomatic (95%) and Ct < 30 (91%) infections, suggesting this could be related to test-seeking behavior.

Consistent with two recent studies^9,14^, we found vaccination to be as effective against the B.1.1.7 variant as non-B.1.1.7 variants. Our study supports this in a broader population, including positives from individuals not reporting symptoms and for the BNT162b2 vaccine in addition to the ChAdOx1 vaccine. Our study had good power to estimate vaccine effectiveness against the B.1.1.7 variant as it was conducted over the period when B.1.1.7 became dominant in the United Kingdom. This is particularly relevant as the variant has now been detected in over 40 countries worldwide^24,25^, and the major phase III vaccine trials were conducted before this strain was dominant^7,8^. We observed a slightly greater reduction in new infection episodes in those vaccinated and aged ≥75 years compared with those <75 years—an effect that was no longer apparent after the second dose, potentially due to the combination of vaccination with reduced social contact in the former group. We currently do not have evidence of the vaccine being less effective in older individuals, as seen elsewhere with natural re-infections^23^, although we would note that, as described above, vaccine effectiveness also includes a non-biological behavioral component and there may be compensation for lower biological activity in older individuals with lower behavioral risk.

There was no evidence of any difference in effectiveness between BNT162b2 and ChAdOx1 vaccines after the first or second doses. However, we cannot exclude the existence of small differences in vaccine effectiveness due to infrequent infections. There are very few direct head-to-head comparisons of both vaccines, especially after second doses, and it is important that differences between separate randomized trials are not directly attributed to the vaccine before considering other differences between trials, including different outcome definitions, populations and circulating SARS-CoV-2 variants^7,8^.

Similar to other studies^8,9,16,18^, we found greater reductions in new positives after two vaccine doses compared with one dose, particularly in infections with self-reported symptoms and low Ct/high viral load. In the United Kingdom, the interval between vaccine doses was extended to 12 weeks to maximize initial coverage and reduce hospitalizations and/or deaths. Our findings highlight the importance of increased protection of individuals receiving the second vaccine dose. Nonetheless, the substantial reduction in positivity after only one dose supports the decision to maximize the initial vaccination coverage.

While some infections, particularly those with Ct ≥ 30, could represent historical infections contracted before vaccination, given the timescales and previous negatives post-vaccination, some will undoubtedly reflect new infections after vaccination. Together with other evidence, this suggests that vaccination does not completely prevent infection following virus exposure, yet minimizes progression to more severe infection^15^. The fact that vaccinated individuals can still be infected, even if it is predominantly with a lower viral burden and/or asymptomatic infections, means that onwards transmission remains a possibility, albeit at lower efficiency^26^. Overall, approximately one-fifth of infections with Ct < 30 were in individuals not reporting symptoms during their episode. These infections could be particularly relevant for transmission as these individuals may not be aware of their infection status despite having a relatively high viral load. Maintaining measures such as social distancing may therefore still be needed to control the virus spread until enough of the population is vaccinated.

We have also shown two vaccine doses to be as effective as previous natural infection. This could be an important consideration during policy development over COVID status certification or so-called COVID passports, and supports considering both previous PCR or serological testing and vaccination data for this^27^.

Looking forward, one key question will be whether immunization offers long-term protection against COVID-19. A recent study showed that the rate of waning and the longevity of neutralizing antibodies varies greatly among individuals with previous COVID-19 infection and suggested that, if similar rates of waning are seen after vaccination or new variants that render vaccines less effective emerge and spread effectively (such as B.1.351 or P.1, which were too rare to assess in our study), more frequent vaccine administration will probably be needed^28^. Overall, we have shown COVID-19 vaccination to be effective in reducing the number of new SARS-CoV-2 infections, with the greatest benefit received after two vaccinations and against symptomatic and high viral burden infections and no difference in effectiveness between the BNT162b2 and ChAdOx1 vaccines.

## Methods

### Study participants

The ONS COVID-19 Infection Survey is a large household survey with longitudinal follow-up (ISRCTN21086382; https://www.ndm.ox.ac.uk/covid-19/covid-19-infection-survey/protocol-and-information-sheets) (details in ref. ^20^). The study received ethical approval from the South Central Berkshire B Research Ethics Committee (20/SC/0195). Private households are randomly selected on a continuous basis from address lists and previous surveys to provide a representative sample across the United Kingdom. For the current analysis, following verbal agreement to participate, a study worker visited each selected household to take written informed consent for individuals aged 2 years and over. Parents or carers provided consent for those aged 2–15 years; those aged 10–15 years also provided written assent. All participants who completed the enrollment visit were offered a £50 voucher, plus one £25 voucher for each further visit. For the current analysis, we only included individuals aged 16 years and over who were potentially eligible for vaccination.

Individuals were asked about demographics, behaviors, work and vaccination uptake (https://www.ndm.ox.ac.uk/covid-19/covid-19-infection-survey/case-record-forms). At the first visit, participants were asked for (optional) consent for follow-up visits every week for the next month, then monthly for 12 months from enrollment. At each visit, enrolled household members provided a nose and throat self-swab following instructions from the study worker. These were comparable to or even more sensitive than swabs performed by healthcare workers^29^. From a random 10–20% of households, those aged 16 years or older were invited to provide blood monthly for antibody testing.

### Laboratory testing

Swabs were couriered directly to the United Kingdom’s national Lighthouse laboratories (Glasgow and the National Biocentre in Milton Keynes (to 8 February 2021)) where samples were tested within the national testing program using identical methodology. The presence of three SARS-CoV-2 genes (*ORF1ab* and the genes transcribing nucleocapsid protein (*N*) and spike protein (*S*)) was identified using RT-PCR with the TaqPath RT-PCR COVID-19 kit (Thermo Fisher Scientific), analyzed using UgenTec FastFinder 3.300.5 (TaqMan 2019-nCoV Assay Kit V2 UK NHS ABI 7500 v2.1; UgenTec). The assay plugin contained an assay-specific algorithm and decision mechanism allowing conversion of the qualitative amplification assay raw data into test results with little manual intervention. Samples were called positive if either *N* or *ORF1ab*, or both, were detected. The *S* gene alone was not considered a reliable positive^29^ but could accompany other genes (that is, one, two or three gene positives).

Blood samples were couriered directly to the University of Oxford, where they were tested for the SARS-CoV-2 antibody using an enzyme-linked immunosorbent assay detecting anti-trimeric spike immunoglobulin G^30^. Before 26 February 2021, the assay used fluorescence detection, as previously described (with a positivity threshold of 8 million units)^3^. After this, it used a commercialized CE-marked version of the assay, the OmniPATH 384 Combi SARS-CoV-2 immunoglobulin G enzyme-linked immunosorbent assay (Thermo Fisher Scientific), with the same antigen and a colorimetric detection system (with a positivity threshold of 42 ng ml^−1^ monoclonal antibody unit equivalents, as determined from 3,840 samples run in parallel).

### Inclusion and exclusion criteria

This analysis included participants aged 16 years or over (that is, those who theoretically could have received vaccination) and all visits with positive or negative swab results from 1 December 2020 to 8 May 2021.

### Vaccination status

Participants were asked about their vaccination status at visits, including the type, number of doses and date(s). Participants from England were also linked to administrative records from the National Immunisation Management Service (NIMS). We used records from NIMS where available. Otherwise, we used records from the survey, since linkage was periodic and NIMS does not contain information about vaccinations received abroad or in Northern Ireland, Scotland and Wales. Where records were available from both NIMS and the survey, agreement on type was 98% and agreement on dates was 95% within ±7 d. The main analysis included any type of COVID-19 vaccination, but there were only sufficient numbers to provide separate estimates for ChAdOx1 and BNT162b2 in analyses that evaluated the results by vaccine type, so other vaccines were excluded from these analyses.

### SARS-CoV-2 infection episodes

PCR-positive results may be obtained at multiple visits after infection, so we grouped positive tests into episodes. Whole-genome sequencing was available on only a subset of positives, and only a subsample provided monthly blood samples for antibody status, so positive episodes were defined using study PCR results. Based on the World Health Organization definition of re-infection as positive tests occurring at least 90 d after the onset of primary infection^31^, but also incorporating multiple consecutive negative tests, we defined the start of a new infection episode as the date of either: (1) the first PCR-positive test in the study (not preceded by any study PCR-positive test by definition); (2) a PCR-positive test after four or more consecutive negative tests; or (3) a PCR-positive test at least 90 d after the start of a previous infection episode, with one or more negative tests immediately preceding this. Positive episodes were used to classify exposure groups and outcomes (see below).

### Exposures

At each study visit, a participant was classified into one of eight different exposure groups based on their current vaccination status, study antibody and PCR tests and (for exposure classification only) positive swab tests linked from the English national testing program^32^ (before visit), as follows:

1. Visits from participants ≥21 d before the first vaccination, including those currently with no vaccination date, with no previous PCR- or antibody-positive result in the study, nor a positive swab test in the national testing program (as defined below) (baseline group)
2. Visits from participants 1–21 d before the first vaccination with no previous PCR- or antibody-positive result in the study, nor a positive swab test in the national testing program
3. Visits 0–7 d following a first vaccination
4. Visits 8–20 d following a first vaccination
5. Visits 21 d or more following a first vaccination (no second dose)
6. Visits after the second vaccination ≥21 d following the first vaccination (post-second dose)
7. Visits from participants who were previously PCR/antibody positive in the study or had a positive swab test in the national testing program; who had a first positive <4 months previously; and who were not (yet) vaccinated
8. Visits from participants who were previously PCR/antibody positive in the study or had a positive swab test in the national testing program; who had a first positive ≥4 months previously; and who were not (yet) vaccinated

We chose these vaccination status categories empirically based on the odds of infection episodes when modeling days since first vaccination as a continuous effect, allowing for nonlinearity by using restricted cubic splines (Extended Data Fig. 1). Exposure group 2 (not vaccinated; not previously positive; 1–21 d before vaccination) was included because there is probably a certain degree of transient reverse causality where individuals are avoiding contact with others before vaccination and vaccination appointments have to be rescheduled if someone tests positive in the weeks before the scheduled visit (21-d cut-off reflecting the period where odds dropped below 0.50 based on Extended Data Fig. 1). Visits from participants who were not vaccinated and who were previously positive were further split by whether the first evidence of the positive test was <4 months ago or longer, because, as not everyone underwent antibody testing, by necessity, our definition of a new positive episode was based on PCR positives only. More positive tests due to intermittent prolonged carriage might be expected for visits with a more recent time since the index positive episode, despite the fact that individuals were considered at risk again for a new positive episode only after having at least four or more consecutive negative tests or at least 90 d since the start of the index positive, with at least one negative study result before the new episode. The 4-month cut-off was arbitrary, being approximately the median time since the first evidence of positivity (median = 125 d). As the antibody status before vaccination was not available for all participants, we defined previous positivity as the participant having either a positive antibody measurement >90 d before the visit or a previous PCR-positive episode. The choice of 90 d was arbitrary but designed to exclude ongoing infections acquired previously being misattributed to current visits. Visits from vaccinated individuals (groups 3–6) were defined irrespective of previous positivity (Supplementary Tables 2 and 4) to reflect the impact of vaccination as being implemented in the United Kingdom (without regard to previous infection). Visits from the same participant were classified in different groups depending on their status at each visit.

### Outcomes

Analysis was based on visits, since these occurred independent of symptoms and were therefore unbiased. Only the first test-positive visit in the first new positive infection episode starting after 1 December was used, dropping all subsequent visits in the same infection episode and all negative visits before the first time a participant could be considered at risk for a new positive episode (as defined above), to avoid misattributing ongoing PCR positivity to visit characteristics and immortal time bias, respectively. Primary analyses included all first new positive infection episodes. Secondary analyses considered infection severity, by classifying positives by Ct value (<30 or ≥30) and self-reported symptoms. The threshold Ct value of 30 was somewhat arbitrary, but corresponds to ~150 copies per ml^26^, and is consistently used in the United Kingdom for many purposes, including algorithms for the review of low-level positives at the Lighthouse laboratories where the PCR tests were performed and a threshold for attempting whole-genome sequencing. For each positive test, a single Ct value was calculated as the arithmetic mean across detected genes (Spearman’s correlation > 0.98), then the minimum value was taken across positives in the infection episode to reflect the greatest measured viral burden within an episode. To allow for presymptomatic positives being identified in the survey, any self-reported symptoms at any visit within 0–35 d after the index positive in each infection episode were included (questions elicited symptoms in the past 7 d at each visit). Finally, positive infection episodes were classified as compatible with the B.1.1.7 SARS-CoV-2 variant (those positive at least once for *ORF1ab* + *N* across the episode and never *S* positive) and those that were incompatible (*ORF1ab* + *N* + *S* or *ORF1ab* + *S* or *N* + *S* at least once across the episode). B.1.1.7 has deletions in the *S* gene leading to *S* gene target failure, and *ORF1ab* + *N* positivity only remains a good proxy for B.1.1.7 from whole-genome sequencing from mid-November 2020^33^. Positives where only a single *N* or single *ORF1ab* gene were detected were excluded from this secondary analysis.

### Confounders

The following potential confounders were adjusted for in all models as potential risk factors for acquiring SARS-CoV-2 infection: geographic area and age in years (see below); sex; ethnicity (white versus non-white as small numbers); index of multiple deprivation (percentile, calculated separately for each country in the United Kingdom)^34–37^; working in a care home; having a patient-facing role in health or social care; the presence of long-term health conditions; household size; multigenerational household; rural–urban classification^38–40^; direct or indirect contact with a hospital or care home; smoking status; mode of travel to work; work location; and visit frequency. Details are shown in Supplementary Table 1.

### Statistical analysis

Associations between the different exposure groups and outcomes (first positive test in an infection episode versus test negative) were evaluated with generalized linear models with a logit link. Robust standard errors were used to account for multiple visits per participant. To adjust for substantial confounding by calendar time and age with nonlinear effects of age, which are also different by region, we included both as restricted cubic splines, with knots at the 20, 40, 60 and 80% percentiles of unique values and interactions between these splines and region/country (regions for England and country for Northern Ireland, Scotland and Wales). Furthermore, given previous observations of different positivity rates by age over time^20^, we added a tensor spline to model the interaction between age and calendar time with the restriction that the interaction was not doubly nonlinear^41^. We considered effect modification by age of vaccination by fitting this same model, but also including an interaction between vaccine exposure group and age <75 versus ≥75 years or long-term health conditions. There were no positives in those aged ≥75 years in one previously infected exposure group, so these groups were excluded from subgroup analysis by age. Pairwise comparisons of the exposure groups were performed using Tukey adjustments for the pairwise comparisons. Analyses were based on complete cases (>99% observations) (Supplementary Table 2). All statistical analyses were performed using standard functions in the following R packages available at https://cran.r-project.org: ggplot2 (version 3.3.2), rms (version 6.0-1), dplyr (version 1.0.2), emmeans (version 1.5.1), haven (version 2.3.1), sandwich (version 3.0-0), ggeffects (version 1.0.1), broom (version 0.7.2), multcomp (version 1.4-14) and Epi (version 2.44).

### Reporting Summary

Further information on research design is available in the Nature Research Reporting Summary linked to this article.

## Online content

Any methods, additional references, Nature Research reporting summaries, source data, extended data, supplementary information, acknowledgements, peer review information; details of author contributions and competing interests; and statements of data and code availability are available at 10.1038/s41591-021-01410-w.

## Supplementary information

Supplementary Information Supplementary Tables 1–10.

Reporting Summary

## Data Availability

Data are still being collected for the COVID-19 Infection Survey. De-identified study data are available for access by accredited researchers in the ONS Secure Research Service (SRS) for accredited research purposes under part 5, chapter 5 of the Digital Economy Act 2017. For further information about accreditation, contact research.Support@ons.gov.uk or visit the SRS website (https://www.ons.gov.uk/aboutus/whatwedo/statistics/requestingstatistics/approvedresearcherscheme).

## Code availability

All statistical analyses were performed using standard functions in the following R packages: ggplot2 (version 3.3.2), rms (version 6.0-1), dplyr (version 1.0.2), emmeans (version 1.5.1), haven (version 2.3.1), sandwich (version 3.0-0), ggeffects (version 1.0.1), broom (version 0.7.2), multcomp (version 1.4-14) and Epi (version 2.44). Code used for data analysis is available upon request.

## References

[1] *Vaccine BNT162b2—Conditions of Authorisation Under Regulation 174* (Medicines and Healthcare Products Regulatory Agency, 2020); https://www.gov.uk/government/publications/regulatory-approval-of-pfizer-biontech-vaccine-for-covid-19/conditions-of-authorisation-for-pfizerbiontech-covid-19-vaccine

[2] *Regulatory Approval of COVID-19 Vaccine AstraZeneca* (Medicines and Healthcare Products Regulatory Agency, 2020); https://www.gov.uk/government/publications/regulatory-approval-of-covid-19-vaccine-astrazeneca

[3] *Regulatory Approval of COVID-19 Vaccine Moderna* (Medicines and Healthcare Products Regulatory Agency, 2021); https://www.gov.uk/government/publications/regulatory-approval-of-covid-19-vaccine-moderna

[4] *Joint Committee on Vaccination and Immunisation: Advice on Priority Groups for COVID-19 Vaccination, 30 December 2020* (Joint Committee on Vaccination and Immunisation, 2020); https://www.gov.uk/government/publications/priority-groups-for-coronavirus-covid-19-vaccination-advice-from-the-jcvi-30-december-2020/joint-committee-on-vaccination-and-immunisation-advice-on-priority-groups-for-covid-19-vaccination-30-december-2020

[5] *Vaccinations in United Kingdom* (Public Health England, 2021); https://coronavirus.data.gov.uk/details/vaccinations

[6] Saad-Roy CM (2021). Epidemiological and evolutionary considerations of SARS-CoV-2 vaccine dosing regimes. Science.

[7] Voysey M (2021). Safety and efficacy of the ChAdOx1 nCoV-19 vaccine (AZD1222) against SARS-CoV-2: an interim analysis of four randomised controlled trials in Brazil, South Africa, and the UK. Lancet.

[8] Polack FP (2020). Safety and efficacy of the BNT162b2 mRNA COVID-19 vaccine. N. Engl. J. Med..

[9] Lumley, S. F. et al. An observational cohort study on the incidence of SARS-CoV-2 infection and B.1.1.7 variant infection in healthcare workers by antibody and vaccination status. Preprint at *medRxiv*10.1101/2021.03.09.21253218 (2021). 10.1093/cid/ciab608 PMC8994591 34216472

[10] Davies NG (2021). Estimated transmissibility and impact of SARS-CoV-2 lineage B.1.1.7 in England. Science.

[11] Davies NG (2021). Increased mortality in community-tested cases of SARS-CoV-2 lineage B.1.1.7. Nature.

[12] Walker, A. S., et al. Increased infections, but not viral burden, with a new SARS-CoV-2 variant. Preprint at *medRxiv*10.1101/2021.01.13.21249721 (2021).

[13] Collier DA (2021). Sensitivity of SARS-CoV-2 B.1.1.7 to mRNA vaccine-elicited antibodies. Nature.

[14] Emary KRW (2021). Efficacy of ChAdOx1 nCoV-19 (AZD1222) vaccine against SARS-CoV-2 variant of concern 202012/01 (B.1.1.7): an exploratory analysis of a randomised controlled trial. Lancet.

[15] Dagan N (2021). BNT162b2 mRNA COVID-19 vaccine in a nationwide mass vaccination setting. N. Engl. J. Med..

[16] Hall VJ (2021). COVID-19 vaccine coverage in health-care workers in England and effectiveness of BNT162b2 mRNA vaccine against infection (SIREN): a prospective, multicentre, cohort study. Lancet.

[17] Bernal JL (2021). Effectiveness of the Pfizer-BioNTech and Oxford-AstraZeneca vaccines on covid-19 related symptoms, hospital admissions, and mortality in older adults in England: test negative case-control study. BMJ.

[18] Thompson MG (2021). Interim estimates of vaccine effectiveness of BNT162b2 and mRNA-1273 COVID-19 vaccines in preventing SARS-CoV-2 infection among health care personnel, first responders, and other essential and frontline workers—eight U.S. locations, December 2020–March 2021. Morb. Mortal. Wkly Rep..

[19] Shrotri, M. et al. Vaccine effectiveness of the first dose of ChAdOx1 nCoV-19 and BNT162b2 against SARS-CoV-2 infection in residents of long-term care facilities (VIVALDI study). Preprint at *MedrXiv*10.1101/2021.03.26.21254391 (2021). 10.1016/S1473-3099(21)00289-9 PMC8221738 34174193

[20] Pouwels KB (2021). Community prevalence of SARS-CoV-2 in England from April to November, 2020: results from the ONS Coronavirus Infection Survey. Lancet Public Health.

[21] Singanayagam A (2020). Duration of infectiousness and correlation with RT-PCR cycle threshold values in cases of COVID-19, England, January to May 2020. Eur. Surveill..

[22] *Book or Manage Your Coronavirus (COVID-19) Vaccination* (NHS, 2021); https://www.nhs.uk/conditions/coronavirus-covid-19/coronavirus-vaccination/book-coronavirus-vaccination/

[23] Hansen CH (2021). Assessment of protection against reinfection with SARS-CoV-2 among 4 million PCR-tested individuals in Denmark in 2020: a population-level observational study. Lancet.

[24] *Global Report Investigating Novel Coronavirus Haplotypes. B.1.1.7* (pangolin, 2021); https://cov-lineages.org/global_report.html

[25] Chaillon, A. & Smith, D. M. Phylogenetic analyses of SARS-CoV-2 B.1.1.7 lineage suggest a single origin followed by multiple exportation events versus convergent evolution. *Clin. Infect. Dis*. 10.1093/cid/ciab265 (2021). 10.1093/cid/ciab265 PMC8083653 33772259

[26] Lee, L. Y. W. et al. SARS-CoV-2 infectivity by viral load, S gene variants and demographic factors and the utility of lateral flow devices to prevent transmission. *Clin. Infect. Dis.* https://doi.org/10.1093/cid/ciab421 (2021). 10.1093/cid/ciab421 PMC8136027 33972994

[27] *Government Asks for Views on COVID-19 Certification* (Cabinet Office, 2021); https://www.gov.uk/government/news/government-asks-for-views-on-covid-19-certification

[28] Chia, W. N. et al. Dynamics of SARS-CoV-2 neutralising antibody responses and duration of immunity: a longitudinal study. *Lancet Microbe*10.1016/S2666-5247(21)00025-2 (2021). 10.1016/S2666-5247(21)00025-2 PMC7987301 33778792

[29] Kojima, N. et al. Self-collected oral fluid and nasal swab specimens demonstrate comparable sensitivity to clinician-collected nasopharyngeal swab specimens for the detection of SARS-CoV-2. *Clin. Infect. Dis*. 10.1093/cid/ciaa1589 (2020). 10.1093/cid/ciaa1589 PMC7665422 33075138

[30] National SARS-CoV-2 Serology Assay Evaluation Group (2020). Performance characteristics of five immunoassays for SARS-CoV-2: a head-to-head benchmark comparison. Lancet Infect. Dis..

[31] *Interim Guidelines for Detecting Cases of Reinfection by SARS-CoV-2* (Pan American Health Organization, 2020).

[32] *NHS Test and Trace Statistics (England): Methodology* (UK Department of Health & Social Care, 2021); https://www.gov.uk/government/publications/nhs-test-and-trace-statistics-england-methodology/nhs-test-and-trace-statistics-england-methodology

[33] *Investigation of SARS-CoV-2 Variants of Concern: Technical Briefings* (Public Health England, 2020); https://www.gov.uk/government/publications/investigation-of-novel-sars-cov-2-variant-variant-of-concern-20201201

[34] *English Indices of Deprivation 2019* (Ministry of Housing, Communities and Local Government, 2019); https://www.gov.uk/government/statistics/english-indices-of-deprivation-2019

[35] *Welsh Index of Multiple Deprivation (Full Index Update with Ranks): 2019* (Statistics for Wales, 2019); https://gov.wales/welsh-index-multiple-deprivation-full-index-update-ranks-2019

[36] *Scottish Index of Multiple Deprivation 2020* (Scottish Government, 2020); https://www.gov.scot/collections/scottish-index-of-multiple-deprivation-2020/

[37] *Northern Ireland Multiple Deprivation Measure 2017 (NIMDM2017)* (Northern Ireland Statistics and Research Agency, 2017); https://www.nisra.gov.uk/statistics/deprivation/northern-ireland-multiple-deprivation-measure-2017-nimdm2017

[38] *Urban–Rural Classification* (Northern Ireland Statistics and Research Agency, 2017); https://www.nisra.gov.uk/support/geography/urban-rural-classification

[39] *Rural Urban Classification* (Department for Environment, Food and Rural Affairs, 2016); https://www.gov.uk/government/collections/rural-urban-classification

[40] *Scottish Government Urban Rural Classification 2016* (Scottish Government, 2018); https://www.gov.scot/publications/scottish-government-urban-rural-classification-2016/pages/2/

[41] Harrell, F. E. Jr. *Regression Modeling Strategies: With Applications to Linear Models, Logistic and Ordinal Regression, and Survival Analysis* (Springer, 2015).

