# Supplementary material for: Impact of vaccination on new SARS-CoV-2 infections in the United Kingdom
Source: Nat Med. 2021 Jun 9;27(8):1370–8. doi: 10.1038/s41591-021-01410-w (PMC8363500; doi:10.1038/s41591-021-01410-w)
Supplement: Supplementary file 1 — Supplementary Tables 1–10. [file 41591_2021_1410_MOESM1_ESM.pdf]

nature portfolio

https://doi.org/10.1038/s41591-021-01410-w

Supplementary information

Impact of vaccination on new SARS-CoV-2 infections in the United Kingdom

In the format provided by the authors and unedited

Supplementary Material

Supplementary Table 1: Characteristics of visits included in analysis

Characteristic [number missing]	Negative, n (%) or median (IQR)	Positive, n (%) or median (IQR)	Total, n (%) or median (IQR)
**Female [0]**	1,034,685 (54)	6,894 (54)	1,041,579 (54)
**White ethnicity [0]**	1,810,206 (94)	11,577 (90)	1,821,783 (94)
**Age [0]**	55 (40, 68)	49 (35, 61)	55 (40, 68)
**Region [0]**			
London	337,701 (17)	3,301 (26)	341,002 (18)
North_West_England	212,234 (11)	1,597 (12)	213,831 (11)
North_East_England	67,594 (3)	473 (4)	68,067 (3)
Yorkshire	148,858 (8)	828 (6)	149,686 (8)
West_Midlands	134,506 (7)	868 (7)	135,374 (7)
East_Midlands	115,518 (6)	752 (6)	116,270 (6)
South_East_England	237,087 (12)	1,509 (12)	238,596 (12)
South_West_England	148,790 (8)	610 (5)	149,400 (8)
East_England	184,003 (10)	1,250 (10)	185,253 (10)
Northern_Ireland	60,847 (3)	361 (3)	612,08 (3)
Scotland	181,273 (9)	744 (6)	182,017 (9)
Wales	103,834 (5)	533 (4)	104,367 (5)
**Household size group [0]**			
One	366,188 (19)	2,016 (16)	368,204 (19)
Two	916,924 (47)	5,072 (40)	921,996 (47)
Three	300,624 (16)	2,436 (19)	303,060 (16)
Four	247,130 (13)	2,283 (18)	249,413 (13)
Five plus	101,379 (5)	1,019 (8)	102,398 (5)
**Multigenerational households [0]**	93,144 (5)	814 (6)	93,958 (5)
**Rural-urban classification [0]**			
Major urban	681,144 (35)	5,789 (45)	686,933 (35)
Urban city/town	817,866 (42)	4954 (39)	822,820 (42)
Rural town	210,277 (11)	1,101 (9)	211,378 (11)
Rural village	222,958 (12)	982 (8)	223,940 (12)
**Deprivation centile (1 = most deprived, 10 = least deprived) [0]**	6 (3, 8)	5 (3, 8)	6 (3, 8)
**Ever reported to be a care home worker [0]**	21,070 (1)	254 (2)	21,324 (1)
**Ever reported to be a patient-facing healthcare worker [0]**	47,633 (2)	471 (4)	48,104 (2)
**Ever reported to be a person-facing social care worker [0]**	21,855 (1)	227 (2)	22,082 (1)
**Ever reported to have a long-term health condition [0]**	524,376 (27)	2,961 (23)	527,337 (27)
**Days since previous visit [0]**			
>14 days	1,496,896 (77)	9,846 (77)	1,506,742 (77)
<=14 days	348,415 (18)	1,960 (15)	350,375 (18)
Enrolment	86,934 (4)	1,020 (8)	87,954 (5)
**Smoking status [0]**			
Non-smoker	1,747,978 (90)	11,655 (91)	1,759,633 (90)
Tobacco smoker	142,392 (7)	841 (7)	143,233 (7)

Only vape	41,875 (2)	330 (3)	42,205 (2)
**Contact with hospital [7503]**			
No	1,499,189 (78)	9,604 (75)	1,508,793 (78)
Yes, I have	266,461 (14)	1,988 (15)	268,449 (14)
No, but someone in HH	159,149 (8)	1,177 (9)	160,326 (8)
**Contact with care home [10814]**			
No	1,857,072 (96)	12,140 (95)	1,869,212 (96)
Yes, I have	38,345 (2)	357 (3)	38,702 (2)
No, but someone in HH	26,098 (1)	245 (2)	26,343 (1)
**Work location/distancing [5759]**			
Working from home	498,588 (26)	3,475 (27)	502,063 (26)
Working elsewhere, easy to maintain 2m	211,157 (11)	1,445 (11)	212,602 (11)
Working elsewhere, relatively easy to maintain 2m	98,223 (5)	854 (7)	99,077 (5)
Working elsewhere, difficult to maintain 2m	79,833 (4)	739 (6)	80,572 (4)
Working elsewhere, very difficult to maintain 1m	135,547 (7)	1,390 (11)	136,937 (7)
Employed but not currently working (including furloughed)	75,289 (4)	949 (7)	76,238 (4)
Not employed	124,245 (6)	935 (7)	125,180 (6)
Student	13,029 (1)	136 (1)	13,165 (1)
Retired	690,610 (36)	2,868 (22)	693,478 (36)
**Work travel method [5796]**			
Not travelling to work	1,389,416 (72)	8,260 (64)	1,397,676 (72)
On foot/bike	76,384 (4)	611 (5)	76,995 (4)
Car/taxi	412,921 (21)	3,435 (27)	416,356 (21)
Train/bus	46,588 (2)	484 (4)	47,072 (2)
Other	1,171 (0)	5 (0)	1,176 (0)

Note: analysis is based on visits rather than participants: factors above and vaccination exposure (detailed in Supplementary Table 2) could change over time. Numbers in square brackets in the first column indicate the number of visits with missing information.

Supplementary Table 2: Vaccination and previous infection status for visits included in analysis

Vaccination and previous infection status	No prior study or national testing programme swab-positive episode and no antibody positive >90 days ago Number of visits (row %) [number of positives]	Prior study or national testing programme swab-positive episode or antibody positive >90 days ago Number of visits (row %) [number of positives]	Total visits (row %) [number of positives]
Not vaccinated, no prior positive, >21 days before vaccination	1,012,808 (100%) [10,721]	0 (0%) [0]	1,012,808 (100%) [10,721]
Not vaccinated, no prior positive, 1-21 days before vaccination	200,553 (100%) [643]	0 (0%) [0]	200,553 (100%) [643]
Vaccinated 0-7 days ago	77,234 (98%) [279]	1,929 (2%) [12]	79,163 (100%) [291]
Vaccinated 8-20 days ago	118,928 (97%) [431]	3,443 (3%) [10]	122,371 (100%) [441]
≥21 days after 1st dose, no second dose	355,639 (96%) [495]	14,520 (4%) [35]	370,159 (100%) [530]
Post second dose	131,554 (96%) [83]	6,021 (4%) [12]	137,575 (100%) [95]
Not vaccinated, previously first positive <4m ago	0 (0%) [0]	10,534 (100%) [76]	10,534 (100%) [76]
Not vaccinated, previously first positive ≥4m ago	0 (0%) [0]	11,908 (100%) [29]	11,908 (100%) [29]

Note: analysis is based on visits rather than participants: factors (Supplementary Table 1) and vaccination exposure could change over time. See methods for definition of swab-positive episodes of infection. As <4% of visits after vaccination occurred after previous infection, these were combined with those not occurring after previous infection to consider the impact of vaccination overall as implemented in the UK.

Supplementary Table 3: Summary of key characteristics by vaccination status

	Not vaccinated, no prior positive, >21 days before vaccination	Not vaccinated, no prior positive, 1-21 days before vaccination	Vaccinated 0-7 days ago	Vaccinated 8-20 days ago	≥21 days after 1st dose, no second dose	Post second dose	Not vaccinated, previously positive <4m ago	Not vaccinated, previously positive ≥4m ago	P-value*
**N (%)**	10721 (84)	643 (5)	291 (2)	441 (3)	530 (4)	95 (1)	76 (1)	29 (0)	
**Minimum Ct, median (IQR)**	28.4 (20.1-32.9)	31.5 (27.5-33.5)	31.5 (20.8-33.8)	30.9 (23.3-33.7)	31.7 (26.8-33.7)	32.7 (27.7-34.0)	32.2 (28.8-34.1)	33.2 (31.0-33.9)	<0.001
**Gene positivity pattern**									
OR+N+S	1705 (16)	60 (9)	22 (8)	25 (6)	25 (5)	2 (2)	7 (9)	4 (14)	
OR+N (B.1.1.7 compatible)	5628 (52)	356 (55)	151 (52)	250 (57)	314 (59)	50 (53)	36 (47)	11 (38)	<0.001
Other single/double	3388 (32)	227 (35)	118 (41)	166 (38)	191 (36)	43 (45)	33 (43)	14 (48)	
**Symptoms**									
None	4492 (42)	286 (44)	165 (57)	236 (54)	323 (61)	79 (83)	54 (71)	26 (90)	
Yes, other	2137 (20)	171 (27)	53 (18)	65 (15)	61 (12)	6 (6)	11 (14)	2 (7)	<0.001
Yes, cough/fever	4083 (38)	184 (29)	71 (24)	138 (31)	145 (27)	10 (11)	10 (13)	1 (3)	
**Ct/ symptoms combination**									
Ct <30 and symptoms reported	4235 (40)	163 (25)	77 (26)	134 (30)	105 (20)	8 (8)	9 (12)	0 (0)	
Ct <30 and no symptoms reported	1788 (17)	84 (13)	51 (18)	68 (15)	96 (18)	19 (20)	14 (18)	5 (17)	
Ct 30+ and symptoms reported	1985 (19)	192 (30)	47 (16)	69 (16)	101 (19)	8 (8)	12 (16)	3 (10)	
Ct 30+ and no symptoms reported	2713 (25)	204 (32)	116 (40)	170 (39)	228 (43)	60 (63)	41 (54)	21 (72)	
Visit with prior negative result			267 (92)	409 (93)	494 (93)	87 (92)			

*p-values calculated using two-sided Kruskal-Wallis test for continuous factors and chi-squared test for categorical factors and indicate differences between each vaccination status group

Note: showing n (col %) or median IQR

Supplementary Table 4: Odds ratios (OR) from main models

Vaccination status		Outcome: All positives		Outcome: split by Ct		Outcome: split by symptoms		Outcome: Split by gene positivity pattern	
		Unadjusted	Adjusted	Mean Ct <30	Mean Ct ≥30	Symptoms reported	No symptoms reported	S gene positive (plus 1 or 2 other genes)	B.1.1.7 compatible (ORFab+N)
**Not vaccinated, no prior positive, 1-21 days before vaccination**	OR (95% CI)	0.30 (0.28, 0.33)	0.28 (0.25, 0.30)	0.21 (0.18, 0.24)	0.35 (0.31, 0.40)	0.29 (0.26, 0.32)	0.27 (0.24, 0.31)	0.25 (0.19, 0.33)	0.28 (0.25, 0.32)
	P-value vs baseline	<0.001	<0.001	<0.001	<0.001	<0.001	<0.001	<0.001	<0.001
**Vaccinated 0-7 days ago**	OR (95% CI)	0.34 (0.31, 0.39)	0.37 (0.32, 0.42)	0.32 (0.26, 0.39)	0.42 (0.36, 0.51)	0.29 (0.24, 0.35)	0.48 (0.40, 0.57)	0.31 (0.20, 0.48)	0.34 (0.28, 0.40)
	P-value vs baseline	<0.001	<0.001	<0.001	<0.001	<0.001	<0.001	<0.001	<0.001
	Pairwise p-value vs group above	0.463	0.002	0.002	0.460	1.00	<0.001	0.993	0.580
**Vaccinated 8-20 days ago**	OR (95% CI)	0.34 (0.31, 0.37)	0.44 (0.39, 0.49)	0.39 (0.33, 0.47)	0.50 (0.42, 0.58)	0.37 (0.31, 0.43)	0.55 (0.46, 0.65)	0.33 (0.21, 0.51)	0.42 (0.36, 0.49)
	P-value vs baseline	<0.001	<0.001	<0.001	<0.001	<0.001	<0.001	<0.001	<0.001
	Pairwise p-value vs group above	1.00	0.251	0.524	0.767	0.396	0.856	1.00	0.347
**≥21 days after 1st dose, no second dose**	OR (95% CI)	0.13 (0.12, 0.15)	0.36 (0.32, 0.41)	0.25 (0.21, 0.31)	0.50 (0.42, 0.59)	0.24 (0.20, 0.30)	0.54 (0.45, 0.64)	0.39 (0.24, 0.63)	0.32 (0.27, 0.38)
	P-value vs baseline	<0.001	<0.001	<0.001	<0.001	<0.001	<0.001	<0.001	<0.001
	Pairwise p-value vs group above	<0.001	0.065	0.001	1.00	0.002	1.00	0.997	0.063
**Post second dose**	OR (95% CI)	0.06 (0.05, 0.08)	0.20 (0.16, 0.26)	0.09 (0.06, 0.15)	0.36 (0.26, 0.50)	0.05 (0.03, 0.10)	0.42 (0.31, 0.57)	0.06 (0.01, 0.32)	0.15 (0.11, 0.21)
	P-value vs baseline	<0.001	<0.001	<0.001	<0.001	<0.001	<0.001	0.001	<0.001
	Pairwise p-value vs group above	<0.001	<0.001	<0.001	0.376	<0.001	<0.001	<0.001	<0.001
	OR (95% CI)	0.68 (0.54, 0.85)	0.72 (0.58, 0.91)	0.39 (0.26, 0.58)	1.17 (0.89, 1.54)	0.33 (0.22, 0.51)	1.34 (1.02, 1.76)	0.53 (0.25, 1.13)	0.58 (0.42, 0.81)

Vaccination status		Outcome: All positives		Outcome: split by Ct		Outcome: split by symptoms		Outcome: Split by gene positivity pattern	
		Unadjusted	Adjusted	Mean Ct <30	Mean Ct ≥30	Symptoms reported	No symptoms reported	S gene positive (plus 1 or 2 other genes)	B.1.1.7 compatible (ORFab+N)
**Not vaccinated, previously positive <4m ago**	P-value vs baseline	0.001	0.006	<0.001	0.272	<0.001	0.035	0.099	0.001
	Pairwise p-value vs group above	<0.001	<0.001	<0.001	<0.001	<0.001	<0.001	0.214	<0.001
**Not vaccinated, previously positive, ≥4m ago**	OR (95% CI)	0.23 (0.16, 0.33)	0.31 (0.21, 0.45)	0.09 (0.04, 0.21)	0.63 (0.42, 0.95)	0.05 (0.02, 0.16)	0.72 (0.49, 1.07)	0.48 (0.22, 1.08)	0.19 (0.10, 0.34)
	P-value vs baseline	<0.001	<0.001	<0.001	0.027	<0.001	0.104	0.075	<0.001
	Pairwise p-value vs second dose	<0.001	0.523	1.00	0.330	1.00	0.338	0.316	0.997

Note: all odds ratios are compared to the baseline (reference) group of **Not vaccinated, no prior positive, >21 days before vaccination.** Results shown graphically in **Figure 2**. P-values vs baseline use a two-sided Wald test vs the baseline group. Pairwise p-values are two-sided and are a Wald test of whether the OR for each vaccine status group is different to the vaccine status group immediately above; so respectively "Vaccinated 0 to 7 days ago, 1 dose" vs "Not vaccinated, no prior positive, 1-21 days before vaccination", "Vaccinated 8 to 20 days ago" vs "Vaccinated 0 to 7 days ago", "Vaccinated ≥ 21 days ago, 2 doses" vs "Vaccinated ≥ 21 days ago, 1 dose" and "Not vaccinated, but positive < or >4m ago" vs "Vaccinated ≥ 21 days ago, 2 doses".

Supplementary Table 5: Odds ratios of other factors in main model for new PCR-positives

Term	Odds ratio	95% CI		P-value
Contact care home				
No	1			
No, but someone in HH	1.33	1.17	1.51	<0.001
Yes, I have	1.33	1.18	1.49	<0.001
Contact hospital				
No	1			
No, but someone in HH	1.11	1.04	1.18	0.075
Yes, I have	1.30	1.24	1.37	<0.001
Vaccination status				
Not vaccinated, no prior positive, >21 days before vaccination	1			
Not vaccinated, no prior positive, 1-21 days before vaccination	0.28	0.25	0.30	<0.001
Vaccinated 0 to 7 days ago	0.37	0.32	0.42	<0.001
Vaccinated 8 to 20 days ago	0.44	0.39	0.49	<0.001
≥21 days since 1st vaccination, no second dose	0.36	0.32	0.41	<0.001
Post second dose	0.20	0.16	0.26	<0.001
Not vaccinated, prior positive <4 months ago	0.72	0.58	0.91	0.006
Not vaccinated, prior positive ≥4 months ago	0.31	0.21	0.45	<0.001
Ethnicity				
White	1			
Non-White	1.13	1.06	1.20	<0.001
Ever care home worker				
No	1			
Yes	1.34	1.16	1.55	<0.001
Ever reported long-term health conditions				
No	1			
Yes	0.99	0.94	1.03	0.524
Ever patient facing healthcare worker				
No	1			
yes	1.51	1.36	1.67	<0.001
Ever person-facing social care worker				
No	1			
Yes	1.46	1.27	1.69	<0.001
Household size				
One	1			
Two	1.00	0.95	1.05	0.994
Three	1.20	1.12	1.28	<0.001
Four	1.34	1.25	1.43	<0.001
Five_plus	1.43	1.32	1.56	<0.001
Deprivation score	0.96	0.95	0.97	<0.001
Multigenerational household				
No	1			

Term	Odds ratio	95% CI		P-value
Yes	0.96	0.89	1.04	0.314
Sex				
Male	1			
Female	0.99	0.96	1.03	0.670
Smoking status				
Non-smoker	1			
Only vape	1.03	0.92	1.15	0.641
Tobacco smoker	0.79	0.73	0.85	<0.001
Days since last visit				
>14 days since last visit	1			
<=14 days since last visit	0.56	0.53	0.59	<0.001
Enrolment	1.01	0.94	1.08	0.754
Work location distancing				
Working from home	1			
Working elsewhere, difficult to maintain 2m	1.32	1.02	1.71	0.037
Working elsewhere, easy to maintain 2m	1.00	0.78	1.29	0.979
Working elsewhere, relatively easy to maintain 2m	1.26	0.97	1.63	0.083
Working elsewhere, very difficult to maintain 1m	1.51	1.17	1.94	0.002
Furloughed/temporarily not working	1.82	1.69	1.96	<0.001
Retired	1.00	0.93	1.08	0.997
Student	1.04	0.82	1.31	0.745
Not working	1.05	0.97	1.13	0.249
Work travel method				
Not travelling to work	1			
Car/taxi	1.20	0.94	1.54	0.148
On foot/bike	1.06	0.82	1.37	0.646
Other	0.51	0.21	1.27	0.151
Train/bus	1.19	0.92	1.54	0.194
Rural urban classification				
Major urban area	1			
Urban city/town	0.85	0.81	0.90	<0.001
Rural town	0.79	0.73	0.85	<0.001
Rural village	0.67	0.62	0.73	<0.001

[1]Interactions included in model: study day by age, study day by region, age by region.

Note: analysis is based on visits rather than participants: factors and vaccination exposure could change over time. Two-sided p-values from Wald test.

Supplementary Table 6: Odds ratios from models split by vaccine type

Vaccination status		Outcome: All positives	Outcome: split by Ct		Outcome: split by symptoms	
		Adjusted	Mean Ct <30	Mean Ct ≥30	Symptoms reported	No symptoms reported
**Not vaccinated, no prior positive, 1-21 days before vaccination**	OR (95% CI)	0.28 (0.25, 0.30)	0.21 (0.18, 0.24)	0.35 (0.31, 0.40)	0.29 (0.26, 0.32)	0.27 (0.24, 0.31)
	P-value vs baseline	<0.001	<0.001	<0.001	<0.001	<0.001
**Vaccinated with ChAdOx1 0-7 days ago**	OR (95% CI)	0.33 (0.27, 0.40)	0.24 (0.17, 0.32)	0.43 (0.34, 0.54)	0.24 (0.18, 0.32)	0.45 (0.35, 0.58)
	P-value vs baseline	<0.001	<0.001	<0.001	<0.001	<0.001
	Pairwise p-value vs group above	0.799	1.00	0.907	0.986	0.006
**Vaccinated with BNT162b2 0-7 days ago**	OR (95% CI)	0.40 (0.34, 0.47)	0.38 (0.30, 0.48)	0.43 (0.34, 0.54)	0.33 (0.26, 0.42)	0.51 (0.40, 0.63)
	P-value vs baseline	<0.001	<0.001	<0.001	<0.001	<0.001
	Pairwise p-value vs group above	0.882	0.269	1.00	0.834	1.00
**Vaccinated with ChAdOx1 8-20 days ago**	OR (95% CI)	0.43 (0.36, 0.50)	0.37 (0.29, 0.48)	0.49 (0.39, 0.61)	0.38 (0.30, 0.49)	0.50 (0.40, 0.64)
	P-value vs baseline	<0.001	<0.001	<0.001	<0.001	<0.001
	Pairwise p-value vs group above	1.00	1.00	0.999	0.998	1.00
**Vaccinated with BNT162b2 8-20 days ago**	OR (95% CI)	0.45 (0.39, 0.51)	0.40 (0.33, 0.49)	0.51 (0.42, 0.61)	0.36 (0.29, 0.44)	0.58 (0.48, 0.70)
	P-value vs baseline	<0.001	<0.001	<0.001	<0.001	<0.001
	Pairwise p-value vs group above	1.00	1.00	1.00	1.00	0.994
**≥21 days after 1st ChAdOx1 dose, no second dose**	OR (95% CI)	0.39 (0.32, 0.46)	0.27 (0.20, 0.35)	0.54 (0.42, 0.68)	0.29 (0.22, 0.38)	0.53 (0.42, 0.67)
	P-value vs baseline	<0.001	<0.001	<0.001	<0.001	<0.001
	Pairwise p-value vs group above	0.94	0.255	1.00	0.976	1.00
**≥21 days after 1st BNT162b2 dose, no second dose**	OR (95% CI)	0.34 (0.29, 0.40)	0.24 (0.19, 0.30)	0.47 (0.39, 0.58)	0.22 (0.17, 0.28)	0.53 (0.43, 0.65)
	P-value vs baseline	<0.001	<0.001	<0.001	<0.001	<0.001
	Pairwise p-value vs group above	0.97	1.00	0.995	0.724	1.00
**Post second ChAdOx1 dose**	OR (95% CI)	0.21 (0.12, 0.35)	0.12 (0.05, 0.28)	0.33 (0.17, 0.65)	0.08 (0.03, 0.22)	0.39 (0.21, 0.73)
	P-value vs baseline	<0.001	<0.001	0.001	<0.001	0.003
	Pairwise p-value vs group above	0.709	0.867	0.996	0.637	0.998
**Post second BNT162b2 dose**	OR (95% CI)	0.20 (0.15, 0.26)	0.08 (0.05, 0.14)	0.37 (0.26, 0.51)	0.05 (0.02, 0.09)	0.42 (0.31, 0.57)
	P-value vs baseline	<0.001	<0.001	<0.001	<0.001	<0.001
	Pairwise p-value vs group above	1.00	0.999	1.00	0.996	1.00
**Not vaccinated, prior positive <4m ago**	OR (95% CI)	0.72 (0.57, 0.91)	0.39 (0.26, 0.58)	1.17 (0.89, 1.54)	0.33 (0.22, 0.51)	1.34 (1.02, 1.75)
	P-value vs baseline	0.006	<0.001	0.271	<0.001	0.036
	Pairwise p-value vs group above	<0.001	<0.001	<0.001	<0.001	<0.001

Vaccination status		Outcome: All positives	Outcome: split by Ct		Outcome: split by symptoms	
		Adjusted	Mean Ct <30	Mean Ct ≥30	Symptoms reported	No symptoms reported
**Not vaccinated, prior positive ≥4m ago**	OR (95% CI)	0.31 (0.21, 0.45)	0.09 (0.04, 0.21)	0.63 (0.42, 0.95)	0.05 (0.02, 0.16)	0.72 (0.49, 1.07)
	P-value vs baseline	<0.001	<0.001	0.027	<0.001	0.102
	Pairwise p-value vs post second BNT162b2 dose	0.704	1.00	0.570	1.00	0.531

Note: all odds ratios are compared to the base category of Not vaccinated, no prior positive, >21 days before vaccination. Results shown graphically in **Figure 4**. P-values vs baseline use a two-sided Wald test vs the baseline group. Pairwise p-values are two-sided and are a Wald test of whether the specified OR vaccine status is different to the vaccine status group above

Supplementary Table 7: Sensitivity analysis for all new PCR-positives excluding potential confounders that are may also be effected by vaccination or prior infection status

Vaccination status	Main model (all covariates)<br><br>OR (95% CI)	Model without 'Work location/ distancing' and 'work travel method'<br><br>OR (95% CI)	Model without 'Work location/ distancing' and 'work travel method' and 'Contact care home'<br>OR (95% CI)
**Not vaccinated, no prior positive, 1-21 days before vaccination**	0.28 (0.25, 0.30)	0.28 (0.26, 0.31)	0.29 (0.26, 0.31)
**Vaccinated 0-7 days ago**	0.37 (0.32, 0.42)	0.38 (0.34, 0.44)	0.39 (0.34, 0.44)
**Vaccinated 8-20 days ago**	0.44 (0.39, 0.49)	0.47 (0.41, 0.52)	0.47 (0.42, 0.53)
**≥21 days after 1st dose, no second dose**	0.36 (0.32, 0.41)	0.39 (0.34, 0.45)	0.40 (0.35, 0.46)
**Post second dose**	0.20 (0.16, 0.26)	0.23 (0.18, 0.30)	0.23 (0.18, 0.30)
**Not vaccinated, prior positive <4m ago**	0.72 (0.58, 0.91)	0.74 (0.59, 0.93)	0.74 (0.59, 0.93)
**Not vaccinated, prior positive ≥4m ago**	0.31 (0.21, 0.45)	0.32 (0.22, 0.46)	0.32 (0.22, 0.46)

Supplementary Table 8: Number of complete cases included in main models

Model	Positive swabs, n (%)	Negative swabs, n (%)	Total, n (%)
**Outcome: All positives**	12,705 (99)	1,915,184 (99)	1,927,889 (99)
**Outcome: Positives based on Ct values**			
Ct <30	6,801 (99)	1,921,088 (99)	1,927,889 (99)
Ct 30+	5,904 (99)	1,921,985 (99)	1,927,889 (99)
**Outcome: Positives based on symptoms**			
Symptoms reported	7,088 (99)	1,920,801 (99)	1,927,889 (99)
No symptoms reported	5,617 (99)	1,922,272 (99)	1,927,889 (99)
**Outcome: Positives based on Ct pattern**			
S gene positive (plus 1 or 2 other genes)	1,941 (99)	1,925,948 (99)	1,927,889 (99)
B.1.1.7 compatible (ORFab+N)	6,734 (99)	1,921,155 (99)	1,927,889 (99)

Supplementary Table 9: Number of participants and visits included in models split by vaccine type

Vaccination status	Number of participants, n (%*)	Positive visits, n (%)	Negative visits, n (%)	Total, n (%)
**Total**	383,812 (100)	12,826 (100)	1,932,245 (100)	1,945,071 (100)
**Not vaccinated, no prior positive, >21 days before vaccination**	329,419 (86)	10,721 (84)	1,002,087 (52)	1,012,808 (52)
**Not vaccinated, no prior positive, 1-21 days before vaccination**	176,296 (46)	643 (5)	199,910 (10)	200,553 (10)
**Vaccinated with ChAdOx1 0-7 days**	50,010 (13)	121 (1)	50,717 (3)	50,838 (3)
**Vaccinated with BNT162b2 0-7 days**	26,734 (7)	168 (1)	27,224 (1)	27,392 (1)
**Vaccinated with ChAdOx1 8-20 days**	73,840 (19)	177 (1)	76,872 (4)	77,049 (4)
**Vaccinated with BNT162b2 8-20 days**	41,004 (11)	261 (2)	43,934 (2)	44,195 (2)
**≥21 days after 1st ChAdOx1 dose, no second dose**	144,859 (38)	236 (2)	222,400 (12)	222,636 (11)
**≥21 days after 1st BNT162b2 dose, no second dose**	81,171 (21)	289 (2)	144,947 (8)	145,236 (7)
**Post second ChAdOx1 dose**	41,018 (11)	21 (0)	47,392 (2)	47,413 (2)
**Post second BNT162b2 dose**	57,646 (15)	72 (1)	89,185 (5)	89,257 (5)
**Not vaccinated, prior positive <4m ago**	8,849 (2)	76 (1)	10,458 (1)	10,534 (1)
**Not vaccinated, prior positive ≥4m ago**	6,468 (2)	29 (0)	11,879 (1)	11,908 (1)
**Other vaccinated administered (not included in model)**	2,173 (1)	12 (0)	5,240 (0)	5,252 (0)

*Percentage of participants does not add to 100 because each participant has multiple visits which can occur in multiple vaccination status categories. For participants, percentages show the percentage of participants contributing any visit to each category.

Supplementary Table 10: Number of participants and visits included in subgroup analyses of all new PCR-positives ny age (A) and long term health conditions (B)

(A) Age

Vaccination status	Age <75				Age 75+			
	Number of participants, n (%*)	Positive visits, n (%)	Negative visits, n (%)	Total visits	Number of participants, n (%*)	Positive visits, n (%)	Negative visits, n (%)	Total visits
**Not vaccinated, no prior positive, >21 days before vaccination**	303874 (79)	10338 (1)	962432 (99)	972770 (100)	25665 (7)	383 (1)	39655 (99)	40038 (100)
**Not vaccinated, no prior positive, 1-21 days before vaccination**	151900 (40)	536 (0)	171516 (100)	172052 (100)	24411 (6)	107 (0)	28394 (100)	28501 (100)
**Vaccinated 0-7 days ago**	66450 (17)	231 (0)	67400 (100)	67631 (100)	11194 (3)	60 (1)	11472 (99)	11532 (100)
**Vaccinated 8-20 days ago**	98597 (26)	340 (0)	103141 (100)	103481 (100)	17270 (4)	101 (1)	18789 (99)	18890 (100)
**≥21 days after 1st dose, no second dose**	193680 (50)	424 (0)	304777 (100)	305201 (100)	33937 (9)	106 (0)	64852 (100)	64958 (100)
**Post second dose**	67700 (18)	50 (0)	88653 (100)	88703 (100)	31622 (8)	45 (0)	48827 (100)	48872 (100)

(B) Long-term health conditions

Vaccination status	Never reported long-term health conditions				Ever reported long-term health conditions			
	Number of participants, n (%*)	Positive swabs, n (%)	Negative swabs, n (%)	Total	Number of participants, n (%*)	Positive swabs, n (%)	Negative swabs, n (%)	Total
**Not vaccinated, no prior positive, >21 days before vaccination**	244816 (64)	8443 (1)	786961 (99)	795404 (100)	84603 (22)	2278 (1)	215126 (99)	217404 (100)
**Not vaccinated, no prior positive, 1-21 days before vaccination**	120409 (31)	418 (0)	136227 (100)	136645 (100)	55887 (15)	225 (0)	63683 (100)	63908 (100)
**Vaccinated 0-7 days ago**	53328 (14)	201 (0)	54117 (100)	54318 (100)	24314 (6)	90 (0)	24755 (100)	24845 (100)
**Vaccinated 8-20 days ago**	78691 (21)	296 (0)	82751 (100)	83047 (100)	37175 (10)	145 (0)	39179 (100)	39324 (100)
**≥21 days after 1st dose, no second dose**	152346 (40)	359 (0)	243036 (100)	243395 (100)	74906 (20)	171 (0)	126593 (100)	126764 (100)
**Post second dose**	62191 (16)	61 (0)	86155 (100)	86216 (100)	37076 (10)	34 (0)	51325 (100)	51359 (100)
**Not vaccinated, prior positive <4m ago**	7345 (2)	62 (1)	8684 (99)	8746 (100)	1504 (0)	14 (1)	1774 (99)	1788 (100)
**Not vaccinated, prior positive ≥4m ago**	5421 (1)	25 (0)	9938 (100)	9963 (100)	1047 (0)	4 (0)	1941 (100)	1945 (100)

* Percentage of participants does not add to 100 because each participant has multiple visits which can occur in multiple vaccination status categories. For participants, percentages show the percentage of participants contributing any visit to each category.
